# Supplementary material for: Effects of Yeast Culture on Laying Performance, Antioxidant Properties, Intestinal Morphology, and Intestinal Flora of Laying Hens
Source: Antioxidants (Basel). 2024 Jun 27;13(7):779. doi: 10.3390/antiox13070779 (PMC11274294; doi:10.3390/antiox13070779)
Supplement: Supplementary file 1 [file antioxidants-13-00779-s001.zip › antioxidants-3055780-supplementary.pdf]

Table S1. The composition of premix.

Ingredients	Content (unit/kg)
Thiamine, mg	1.6
Riboflavin, mg	6.5
Niacin, mg	28
Choline chloride, mg	450
Calcium pantothenate, mg	9
Pyridoxin, mg	3.25
Biotin, mg	0.07
Folic acid, mg	0.8
Cobalamin, mg	0.015
Iron (Fe), mg	267.5
Copper (Cu), mg	13
Manganese (Mn), mg	95
Zinc (Zn), mg	90
Iodine (I), mg	1.35
Se (mg)	0.29
Vitamin A, IU	3000
Vitamin D_3, IU	2400
Vitamin E, IU	16
Vitamin K_3, mg	2

Table S2. Effect of yeast culture (YC) supplementation on serum antioxidant capacity of laying hens [1].

Items	Control	YC1.0	YC2.0	SEM	*p* value
T-AOC (U/mg protein)	9.30 ± 1.26[b]	12.64 ± 0.83[a]	13.80 ± 1.41[a]	0.584	＜0.001
SOD (U/mg protein)	546.42 ± 55.20	582.23 ± 78.06	611.22 ± 64.24	17.403	0.337
GSH-PX (U/ml)	1961.60 ± 96.94[b]	2274.40 ± 199.93[a]	2336.80 ± 212.63[a]	61.043	0.013
MDA (nmol/L)	6.24 ± 1.00[a]	3.76 ± 0.46[b]	3.46 ± 0.73[b]	0.379	＜0.001

[1] Data are means of 5 replicates of 20 samples each replicate. SEM, standard error of mean. [a, b] Labeled in a row, the difference is significant (*p<0.05*), and labels contain the same lowercase letters or no letters, the differences are not significant (*p>0.05*).

Table S3. The Effect of yeast culture (YC) supplementation on relative abundance of cecal microflora at phylum and genus levels of laying hens [1].

Items		Control	YC1.0	YC2.0	SEM	*p* value
Phylum level	Bacteroidetes	62.53 ± 4.51	65.11±4.01	69.73±5.34	1.368	0.084
	Firmicutes	33.11 ± 4.35	29.74±3.81	25.71±5.30	1.352	0.070
	Actinobacteria	0.95 ± 0.13[a]	2.15±0.37[b]	0.62±0.29[a]	0.188	＜0.001
	Proteobacteria	0.83 ± 0.22[a]	0.44±0.20[b]	0.58±0.14[ab]	0.063	0.022

	Bacteroidales	20.30 ± 3.08[b]	25.53±3.64[ab]	28.74±3.72[a]	1.250	0.008
	Bacteroides	22.43 ± 2.53	18.19±5.40	17.04±4.08	1.173	0.142
	Unclassified_S24-7	6.59 ± 1.87	9.40±1.91	8.62±2.35	0.585	0.124
	Ruminococcaceae	9.54 ± 1.91	9.18±1.04	8.16±3.12	0.547	0.599
	Unclassified_BS11	5.38 ± 1.30[ab]	4.36±1.56[b]	7.73±1.86[a]	0.535	0.016
	Clostridiales	5.39 ± 0.82	4.58±0.95	4.34±1.48	0.294	0.333
	Lactobacillus	2.58 ± 1.46	3.66±1.20	4.38±1.32	0.375	0.142
Genus level	*Veillonellaceae*	3.90 ± 0.70[a]	2.09±0.94[b]	1.35±0.42[b]	0.334	＜0.001
	Prevotella	1.86 ± 0.34	1.83±0.62	1.18±0.49	0.145	0.089
	Parabacteroides	1.69 ± 0.44	1.46±0.50	1.72±0.62	0.130	0.703
	Coriobacteriaceae	1.78 ± 0.86[a]	1.92±0.66[a]	0.47±0.16[b]	0.232	0.006
	Lachnospiraceae	1.52 ± 0.54	1.92±1.15	1.00±0.62	0.220	0.242
	Faecalibacterium	1.81 ± 0.71	1.52±1.01	1.32±0.44	0.189	0.609
	Rikenellaceae	0.99 ± 0.39	1.60±0.51	1.22±0.57	0.136	0.192
	Oscillospira	1.53±0.36[a]	0.85±0.21[b]	1.69±0.47[a]	0.130	0.008
	Ruminococcus	2.59±0.41[a]	1.23±0.65[b]	0.79±0.27[b]	0.233	＜0.001

[1] Data are means of 5 replicates of 20 samples each replicate. SEM, standard error of mean. a, b Labeled in a row, the difference is significant (*p<0.05*), and labels contain the same lowercase letters or no letters, the differences are not significant (*p>0.05*).
